# Glucose homeostasis during recurrent periods of sleep restriction and recovery in healthy young adults

**DOI:** 10.1093/sleep/zsaf339

**Published:** 2025-10-30

**Authors:** Yuki Y Y Cheung, Torance Y L Tan, Tiffany B Koa, Chin Meng Khoo, June C Lo

**Affiliations:** Centre for Sleep and Cognition, Yong Loo Lin School of Medicine, National University of Singapore, Singapore, Singapore; Centre for Sleep and Cognition, Yong Loo Lin School of Medicine, National University of Singapore, Singapore, Singapore; Centre for Sleep and Cognition, Yong Loo Lin School of Medicine, National University of Singapore, Singapore, Singapore; Department of Medicine, Yong Loo Lin School of Medicine, National University of Singapore, Singapore, Singapore; Centre for Sleep and Cognition, Yong Loo Lin School of Medicine, National University of Singapore, Singapore, Singapore; Department of Medicine, Yong Loo Lin School of Medicine, National University of Singapore, Singapore, Singapore; Human Potential Translational Research Programme, Yong Loo Lin School of Medicine, National University of Singapore, Singapore, Singapore

**Keywords:** glucose, insulin, intraindividual variability, metabolism, sleep restriction, sleep variability

## Abstract

**Study Objectives:**

To investigate if glucose homeostasis was impaired during recurrent periods of sleep restriction and recovery, and if the impairment was moderated by sleep duration variability across nights.

**Methods:**

In this 16-day laboratory-based study, 48 healthy young adults underwent two baseline nights of 8-h time-in-bed (TIB), followed by two cycles of “weekday” sleep opportunity manipulation (control group’s TIB: 8-h/night; stable short sleep group: 6-h/night; variable short sleep group: 8-, 4-, 8-, 4-, and 6-h from the first to fifth night) and “weekend” recovery (all groups: 8-h/night). Plasma glucose and insulin concentrations during fasting and oral glucose tolerance tests (OGTTs) were measured at the end of baseline and each manipulation period.

**Results:**

No significant group×day interaction on glucose or insulin outcomes was found (*p* > .15). At 2-h post-glucose load, the control group’s glucose levels remained relatively stable in all OGTTs (*p* > .08), although their insulin levels increased from baseline (*p* < .02) probably due to sedentariness in the laboratory. The stable short sleep group also showed increased insulin levels in the first week (*p* = .02), but their glucose levels still increased from baseline (*p* = .02; Cohen’s *d_z_* = 0.39). Importantly, in both weeks of sleep restriction, the variable short sleep group failed to significantly elevate their insulin levels (*p* > .30); hence, their glucose levels increased from baseline (*p* < .01, *d_z_* ≥ 0.99) more prominently than the stable short sleep group.

**Conclusions:**

Glucose homeostasis was impaired in healthy young adults after recurrent periods of sleep restriction. Variable and stable short sleep schedules may impair glucose tolerance to different extents and in different pathways.

**Clinical Trial:**

Performance, Mood, and Brain and Metabolic Functions During Different Sleep Schedules (STAVAR), https://www.clinicaltrials.gov/study/NCT04731662, NCT04731662.

**Statement of Significance**

Simulating two consecutive work weeks, our study showed that weekday sleep restriction led to decreased glucose tolerance despite intervening weekend recovery sleep. The extents and presentations of glucose tolerance depended on the variability in night-to-night sleep duration: the stable short sleep group experienced early signs of insulin resistance with compensatory insulin hypersecretion and small elevation in glucose concentrations, while the variable short sleep group experienced possible beta cell functional impairment, resulting in an absence of significant compensatory insulin hypersecretion and prominent increases in glucose levels. Critically, having a time-in-bed within the age-specific recommended range every night appeared to be the only way to optimize glucose tolerance, thereby minimizing the risk for type 2 diabetes mellitus.

## Graphical Abstract

**Graphical Abstract:**
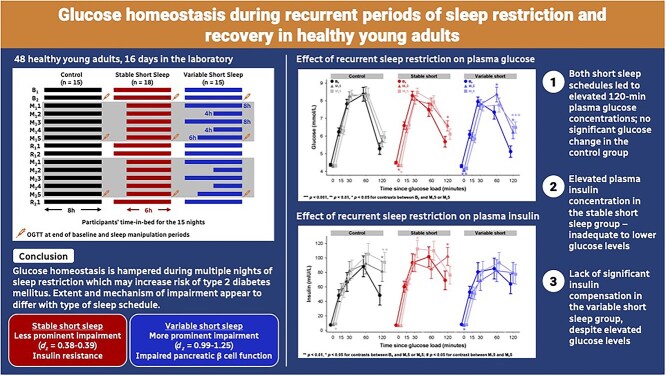

## Introduction

In modern societies, short sleep is common in adults, with about one-fourth of the population sleeping less than the minimum age-specific recommended duration of 7 h [1–4]. Short sleep duration is associated with a higher risk of type 2 diabetes mellitus (DM) [5]. DM is one of the most challenging health problems in the 21st century, with a global prevalence that has increased from 4.6% (151 million people) in 2000 to 10.5% (536.6 million people) in 2021, and the prevalence of DM is estimated to increase further to 12.2% (783.2 million people) in 2045 [6, 7]. In 2010, it was estimated that the annual global health expenditure on diabetes was at least USD 376 billion, which was equivalent to 12% of total health expenditure [8].

Type 2 DM is characterized by chronic hyperglycemia. One of the predominant pathophysiology defects is insulin resistance, and abundant evidence shows that short sleep duration reduces insulin sensitivity [9–17]. In experimental studies, glucose tolerance or insulin sensitivity was impaired when time-in-bed (TIB) was limited to 4 to 5.5 h for 1 to 14 nights [9, 10, 12–14, 18–22]. However, the TIBs used in these studies were relatively short and unlikely to last for prolonged periods in real life [4]. The findings were inconsistent among the few studies investigating the effect of milder sleep restriction. Specifically, while reduced insulin sensitivity was found after three nights of TIB reduction for 1 to 3 h to a mean TIB of 6.0 h in non-obese young adults [17], insulin sensitivity did not change in healthy young adults after their TIB had been reduced for 1.7 h to 5.8 h on average for one night [23], or for 1.5 h to a mean TIB of 6.3 h for 3 weeks [24].

Since sleep curtailment typically occurs on weekdays, and individuals often extend their sleep on weekends [4, 25] some experimental studies have examined the benefits of recovery sleep for glucose homeostasis. Among young men with a history of restricted sleep during the work week and regular weekend catch-up sleep for the past 6 months, glucose tolerance and insulin sensitivity were better after a three-night weekend of 10-h catch-up sleep compared to continuing 6-h sleep restriction [26]. Also, recovery sleep opportunities after 3 weeks of sleep restriction to 5.6 h per 24 h (together with circadian disruption in a forced desynchrony protocol) have been shown to reduce post-prandial glucose level [27]. Nevertheless, epidemiological studies have reported that extending (and delaying) sleep on weekends relative to weekdays, i.e., social jetlag, is associated with an increased risk of type 2 DM [28–33].

In addition, some studies have provided insights into the effect of weekend recovery sleep on glucose metabolism after weekday sleep restriction. Following four nights of 4- to 5-h TIB, 2 nights of 8- to 12-h TIB [34, 35], or even just 1 night of 10-h TIB [36], was sufficient for full recovery of insulin sensitivity to the baseline level. Of note, Depner et al. found that ad libitum weekend recovery sleep of a total of 1.1 h above baseline did not prevent reduced insulin sensitivity when participants were exposed to the second period of sleep restriction in their study [37]. However, glucose metabolic responses during recurrent weeks of weekday sleep restriction and weekend recovery sleep remain to be systematically investigated.

Furthermore, it is important to note that recovery sleep does not only take place over the weekend, but can also occur on weekdays. Such sleep schedules increase intraindividual variability in weekday sleep duration, which is more pronounced in Asian societies than in Europe, North America, and Oceania probably because the Asian work culture delays bedtime and shortens sleep duration, which in turn leads individuals to catch up on sleep whenever they can rather than wait until the end of the week [4]. Sleep variability has been linked with various suboptimal health outcomes in epidemiological studies [38, 39]; however, in our recent laboratory-based study, when healthy young adults underwent two weeks of sleep restriction on simulated weekdays and recovery sleep on simulated weekends, a variable short sleep schedule that allowed for prophylactic and/or recovery sleep opportunities on weekdays (TIB from the first to the fifth night: 8, 4, 8, 4, and 6 h) was found to induce less vigilance deficits as compared a stable short sleep schedule of the same total TIB across nights (five nights of 6-h TIB) [40]. Whether variable and stable short sleep have similar impacts on glucose tolerance is unknown.

To address these knowledge gaps in the literature, this study aimed to investigate glucose homeostasis during recurrent periods of sleep restriction on weekdays and recovery on weekends—a typical sleep pattern among adults in many countries [4, 25], and whether among sleep-restricted individuals, glucose homeostasis would depend on the variability in sleep duration across weeknights.

## Materials and Methods

### Participants

Fifty-nine young adults (24 males) participated in the study. All participants had to be between 21 and 35 years of age and had to have a body mass index (BMI) between 18.5 and 24.9 kg/m^2^. Exclusion criteria included (1) history of sleep disorders, chronic physical illnesses or mental disorders, (2) being a habitual short sleeper (self-reported daily average TIB < 6 h), (3) having an extreme chronotype [41], (4) smoking, (5) daily consumption of >4 cups of caffeinated beverages, (6) weekly consumption of >13 units of alcohol, (7) being a shift worker, (8) traveling across >2 time zones one month before the study, and (9) being pregnant or planning for pregnancy. Owing to the need for blood draw and oral glucose tolerance tests (OGTTs) in the study, participants were also excluded if they (1) had trypanophobia, or (2) had experienced dizziness or had fainted in any previous blood draws or cannulations. Further details of recruitment and screening criteria have been published [40].

**Figure 1:**
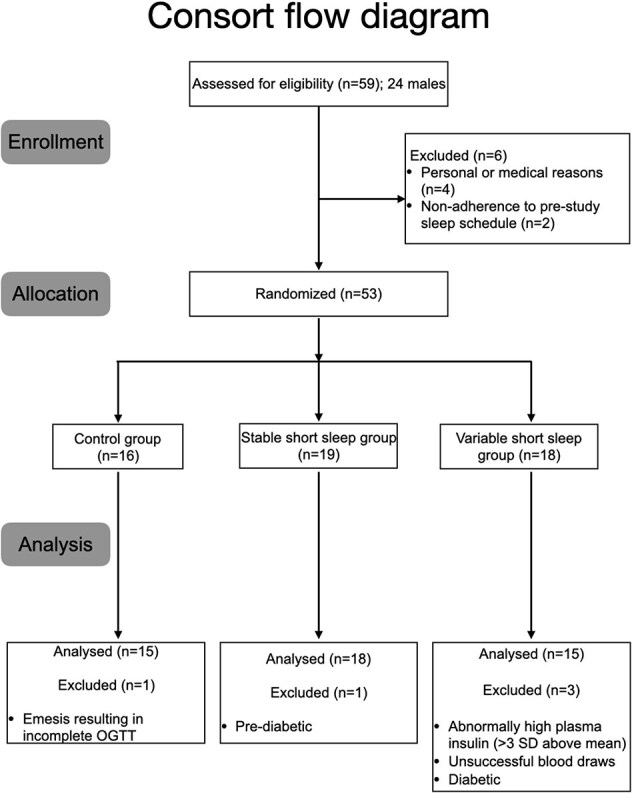
CONSORT flow diagram depicting the flow of participants from enrolment to analysis with participant exclusion documented and accounted for.

Four participants withdrew before the start of the study due to personal or medical reasons, and two participants were excluded on the first day of the study due to non-adherence to their assigned sleep schedules one week before the study (Figure 1). Moreover, data from five participants were excluded from statistical analyses: two due to impaired glucose tolerance suggested by 2-h plasma glucose concentration >7.8 mmol/L in baseline OGTT, one due to unsuccessful blood draws, one due to difficulty in consumption of the glucose solution, and one due to abnormally high plasma insulin concentrations (area under curve being 3 standard deviations above the mean in all three OGTTs). Therefore, 48 participants were included in the final analysis (Figure 1).

This study was approved by the Institutional Review Board of the National University of Singapore. Informed written consent was obtained from all participants. Participants were financially reimbursed for their participation.

### One-week pre-study protocol

One week before the study, participants were required to adhere to a daily 8-h sleep schedule and to wake up at their self-reported habitual wake time. This allowed for circadian entrainment and minimized the effects of prior sleep loss during the study. Compliance was monitored by actigraphy (Actiwatch 2, Philips Respironics, Inc., Murrysville, PA, USA) and sleep diaries. Participants were instructed not to take any naps or consume any food and beverages containing alcohol or caffeine for the three days leading up to the study.

### 16-day study protocol

**Figure 2:**
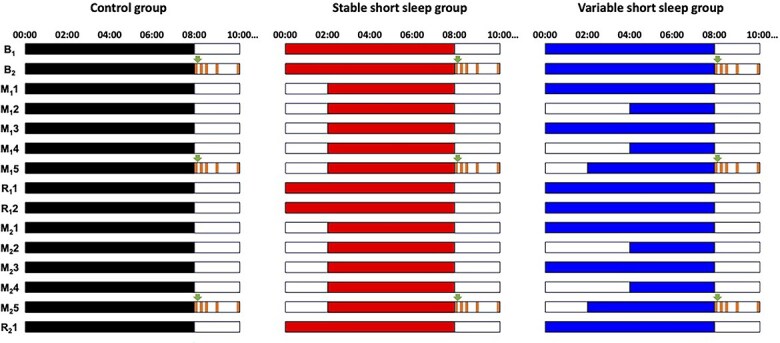
Study protocol. In this 16-day study, all the participants started with two baseline nights (B_1_ and B_2_) with 8-h TIB. This was followed by the first cycle of sleep opportunity manipulation for five nights (M_1_1 to M_1_5; control group’s TIB: 8 h/night; stable short sleep group: 6 h/night; variable short sleep group: 8, 4, 8, 4, and 6 h from the first to the fifth manipulation night) and two nights of recovery sleep (R_1_1 and R_1_2; TIB for all groups: 8 h/night). The second cycle consisted of another five nights of TIB manipulation (M_2_1 to M_2_5) and one night of recovery sleep (R_2_1). Black, red, and blue bars indicate the scheduled sleep periods of the control group, the stable short sleep group, and the variable short sleep group respectively. Lights-on time was aligned to each participant’s habitual wake times and were kept constant throughout the protocol, with shorter TIBs achieved by delaying lights-off time, i.e., bedtimes. Sleep timings illustrated here are based on a lights-on time of 08:00. On days B_2_, M_1_5, and M_2_5, OGTT were performed. Green arrows indicate glucose drink consumption, which took place from 15 to 30 min after wake time. Orange boxes indicate blood collection, which was performed before glucose drink consumption at a fasting state (0 min), as well as 15, 30, 60, and 120 min after drink consumption.

The study lasted 16 days (Figure 2) and was conducted at the Centre for Sleep and Cognition, National University of Singapore. Each participant was assigned a sound-attenuated sleep room without windows. Participants stayed in our research facilities and were continuously monitored by the research staff. Except during scheduled experimental tasks, meal times and sleep periods, they were encouraged to spend their time in a common area where they were exposed to both natural and artificial light. Video gaming was prohibited during the evening to prevent any possible effects on sleep. Participants were also prohibited from napping, consuming snacks, or performing strenuous physical activity.

Participants were randomly assigned to one of the three groups—control group, stable short sleep group, or variable short sleep group. The study began with two baseline nights (B_1_ and B_2_) of 8-h TIB for all participants for adaptation and baseline measurement. This was followed by two cycles of five-night sleep opportunity manipulation and one- or two-night recovery sleep (Figure 2). During the two manipulation periods (M_1_1 to M_1_5 and M_2_1 to M_2_5), TIB for the control group and the stable short sleep groups was 8 and 6 h per night respectively, while TIB for the variable short sleep group was 8, 4, 8, 4, and 6 h from the first to the fifth night. Thus, during the manipulation periods, the total TIB for the two short sleep groups was identical (i.e., 30 h), but variability in TIB across nights differed between the groups. During the recovery sleep periods (R_1_1 to R_1_2 and R_2_1), all participants were provided with an 8-h sleep opportunity each night. Participants were scheduled to wake up at their habitual wake time throughout the study, and hence, bedtime was delayed accordingly for the nights with shorter sleep opportunities.

Participants’ sleep was measured by polysomnography (PSG) every night. The current work reports the TIB and total sleep time (TST) findings from the 48 participants included in the OGTT analyses. The TIB, TST, and sleep macro- and micro-structure data from a larger sample of 52 individuals were previously reported [40, 42]. At the end of the baseline period and each of the sleep opportunity manipulation periods (days B_2_, M_1_5, and M_2_5), an OGTT was performed in the morning. To minimize confounding effects, the diet was strictly controlled and designed to achieve zero weight gain or loss at the end of the study. Caloric intake was specific to each participant, calculated based on their daily caloric requirement according to age, sex, height, weight, and level of physical activity, which is sedentary in this situation due to the restriction of strenuous physical activity throughout the study [43]. Each meal contained 50% carbohydrates, 15% protein, and 35% fat.

### Oral glucose tolerance test

All participants had undergone overnight fasting for at least 8 h before the test. Upon 15 to 30 min after waking up, an intravenous catheter was inserted into the participant’s vein in the forearm. Blood samples were collected before (0 min) and 15, 30, 60, and 120 min after 75 g glucose solution consumption, using antiglycolytic-containing and serum-separator tubes, and processed by the National University Hospital Referral Laboratories. Blood samples were centrifuged using horizontal rotor centrifuges operated at 3000 rpm for 8 min at room temperature before analysis. Due to a change of instruments at the National University Hospital Referral Laboratories midway through data collection, plasma glucose concentrations were analyzed under Beckman Coulter AU 5800 using an enzymatic-based methodology from February 18, 2021 to February 25, 2022 and Abbott Alinity C using an enzymatic-based methodology (Hexokinase/G-6-PDH) from February 26, 2022 to December 21, 2022. Plasma insulin concentrations were analyzed under the Access Ultrasensitive Insulin assay kit using a sandwich immunoassay methodology from February 18, 2021 to February 25, 2022 and the Abbott Alinity I insulin assay kit using a chemiluminescent microparticle methodology from February 26, 2022 to December 21, 2022.

Area under the curve (AUC) for plasma glucose and insulin concentrations throughout OGTT was calculated using the trapezoidal rule. We also derived the Matsuda Index, which was designed to indicate values similar to the rate of disappearance of plasma glucose measured by insulin clamp, to measure and quantify insulin sensitivity [44]. Finally, the insulinogenic index (IGI) at 30 min during the OGTTs was computed to estimate the acute insulin secretion after glucose loading and serves as an indicator of beta cell function [45].

### Polysomnography

Electroencephalography (EEG) was performed using a SOMNOtouch recorder (SOMNOmedics GmbH, Randersacker, Germany) on three channels—C3, F3, and O1 in the International 10–20 system, with the contralateral mastoid (A2) used as reference. On rare occasions, such as when a participant showed minor skin sensitivity at the electrode placement site following multiple days of PSG application, channels C4, F4, and O2 would be used instead, with A1 as the reference. Electrodes placed at Cz and Fpz were used as common reference and ground electrodes, respectively. Electrooculography (EOG) and submental electromyography (EMG) were also used. Impedance was kept below 5 kΩ for EEG and 10 kΩ for EOG and EMG electrodes. Signal was sampled at 256 Hz and filtered between 0.2 and 35 Hz for EEG, and between 0.2 and 10 Hz for EOG. Sleep stages and artifactual epochs were automatically scored using an automatic PSG-scoring software (Neurobit PSG, Neurobit Inc., NY, USA) [46] in conjunction with the FASST toolbox (http://www.montefiore.ulg.ac.be/∼phillips/FASST.html), and visually checked by trained technicians, following criteria set by the American Academy of Sleep Medicine Manual for the Scoring of Sleep and Associated Events [47]. Pulse oximetry was used on the first night (B_1_) to screen for undiagnosed sleep apnea.

### Statistical analysis

Statistical analyses were conducted using SAS software 9.4 (SAS Institute Inc., Cary, NC, USA). To investigate whether the three groups differed in any of the screening parameters, one-way ANOVAs or chi-squared tests were performed. To determine the effectiveness of our TIB manipulation, we used general linear mixed models with PROC MIXED to ascertain the effects of group, day (from night B_2_ to R_2_1), and group x day interaction on TIB and TST. In addition, for each sleep opportunity manipulation period, to test whether the stable and the variable short sleep groups differed in the amount of sleep they obtained, we averaged the TST across the five manipulation nights (M_1_1 to M_1_5 and M_2_1 to M_2_5) for each group and used independent-samples t tests to investigate potential group differences. PSG data from night B_1_ were not included in any of the PSG analyses to avoid possible first-night effects [48].

To determine the effects of group, day (B_2_, M_1_5, and M_2_5), and group x day interaction on glucose tolerance and insulin secretion/sensitivity, general linear mixed models with PROC MIXED were performed on the AUC measures, plasma glucose and insulin concentrations at each sampling time point, Matsuda Index, and IGI. Means, standard errors, and 95% CIs are presented unless otherwise specified. Since our sample might be considered small, we quantified the overall effects of group and day, as well as their interaction on glucose and insulin outcomes, with Cohen’s *ƒ*^2^, which were calculated as *ƒ*^2^ = (*u*/*v*) × *F*, with *u* and *v* respectively being the numerator and denominator degrees of freedom of the *F* statistic used to determine the corresponding main or interaction effect in the general linear mixed model analysis [49]. The cutoffs for small, medium, and large effect sizes were 0.02, 0.15, and 0.35, respectively [49]. To quantify within-subject changes across days into the protocol, we derived Cohen’s *d_z_*, with 0.20, 0.50, and 0.80 as cutoffs for small, medium, and large effects [49].

## Results

### Sample characteristics

There were no statistically significant group differences in age, sex, BMI, consumption of alcoholic or caffeinated beverages, daytime sleepiness, severity of insomnia, morningness-eveningness preference, habitual sleep timing and duration, or sleep quality and disturbances (*p* > .21; Table 1).

**Table 1:** Demographics and habitual sleep characteristics for all groups

	Control group		Stable short sleep group		Variable short sleep group			
	Mean	SD	Mean	SD	Mean	SD	*F*/*ꭓ*^2^	*p*
*n*	15	–	18	–	15	–	–	–
Age (y)	23.00	1.81	22.78	1.77	22.67	1.54	0.15	.86
Sex (% males)	53.33	–	50.00	–	46.67	–	0.13	.94
BMI (kg/m^2^)	21.36	2.06	20.86	1.65	20.61	2.20	0.57	.57
Caffeinated drinks per day (cups)	0.78	1.07	0.63	0.77	0.79	0.41	0.22	.81
Alcohol consumed per week (units)	0.90	1.80	0.85	1.22	0.58	1.83	0.17	.84
ESS score	4.73	2.89	6.06	3.59	5.87	3.60	0.70	.50
ISI score	3.53	2.53	4.28	3.29	2.80	2.93	1.02	.37
MEQ score	48.27	8.29	47.39	5.77	46.07	6.56	0.39	.68
PSQI								
Average TIB (h)	8.24	0.96	7.90	0.65	7.98	0.62	0.90	.42
Average TST (h)	7.74	0.95	7.28	0.61	7.58	0.60	1.64	.21
Bedtime (clock time)	00:18	01:00	00:38	00:47	00:44	01:08	0.82	.45
Wake time (clock time)	08:33	01:06	08:32	00:56	08:43	01:01	0.16	.85
Global score	2.53	1.68	3.33	1.71	2.67	1.50	1.15	.33

Abbreviations: BMI, body mass index; ESS, Epworth Sleepiness Scale; ISI, Insomnia Severity Index; MEQ, Morningness-Eveningness Questionnaire; PSQI, Pittsburgh Sleep Quality Index; SD, standard deviation; TIB, time in bed; TST, total sleep time.

### Sleep duration during the protocol

**Figure 3:**
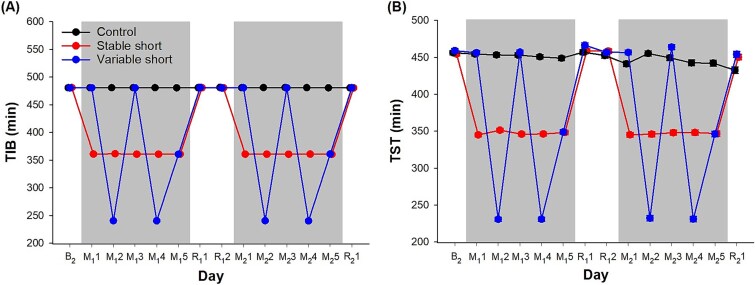
Polysomnographically assessed (A) TIB and (B) TST of the three groups. Least square means and standard errors (negligible) derived from general linear mixed models are depicted for the control group (black), the stable short sleep group (red), and the variable short sleep group (blue) from the second baseline night (B_2_) to the first and second cycles of sleep opportunity manipulation (M_1_1 to M_1_5 and M_2_1 to M_2_5) and recovery (R_1_1 to R_1_2 and R_2_1). Shaded gray areas indicate the sleep manipulation periods.

Our sleep opportunity manipulation was successful as evidenced by the significant group × day interaction on TIB (*F* = 93 286.60, *p* < .001). The negligible error bars in Figure 3A demonstrated that there were minimal inter-individual differences in TIB each night within each of the three groups.

There was also a significant group × day interaction on TST (*F* = 502.84, *p* < .001; Figure 3B). At baseline, TST of all three groups was just above 456 min (*p* > .23). The control group’s mean TST remained between 441.2 and 457.0 min for the rest of the protocol. For the stable short sleep group, mean TST was significantly reduced to 345.1–351.4 min during the two sleep manipulation periods relative to baseline (*p* < .001). For the variable short sleep group, as compared to baseline, mean TST during the manipulation periods was significantly reduced to around 230.7–232.3 min during the 4-h TIB nights (*p* < .001), around 346.3–348.9 min during the 6-h TIB nights (*p* < .001), and remained at around 456.3–463.9 min during the 8-h TIB nights (*p* > .25). Importantly, during each sleep manipulation period, the TST averaged across the five manipulation nights did not significantly differ between the stable and the variable short sleep groups (M_1_1 to M_1_5: *p* = .23, M_2_1 to M_2_5: *p* = .71), indicating that both short sleep groups slept for similar durations.

During the recovery nights, TST of both the stable and the variable short sleep groups returned to 450.4–466.5 min, all of which were similar to baseline TST (*p* > .08).

### Plasma glucose profile

**Figure 4:**
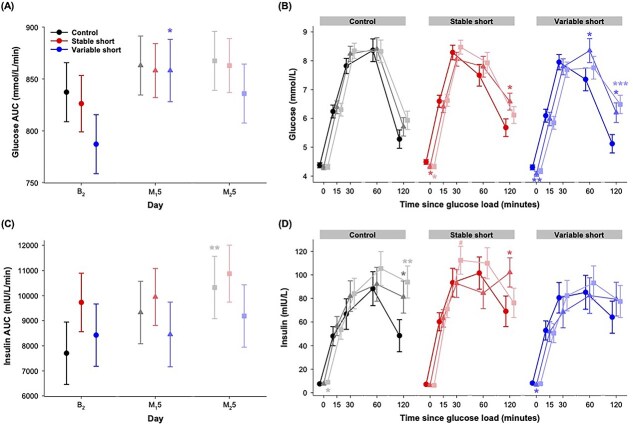
Effects of different sleep schedules on plasma glucose and insulin profiles during OGTT. Mean and standard errors of (A) AUC of plasma glucose concentration, (B) plasma glucose concentration at all time points, (C) AUC of plasma insulin concentration, and (D) plasma insulin concentration at all time points are plotted for the control group (black), the stable short sleep group (red), and the variable short sleep group (blue) on the second baseline day (B_2_ [circle]), and the day after the last sleep opportunity manipulation night in the first and second cycle (M_1_5 [triangle] and M_2_5 [square]). Asterisks indicate significant difference from day B_2_ for each group (*p < .05; **p < .01; ***p < .001), and hashtag indicates significant difference between M_1_5 and M_2_5 (#*p* < .05).

The main effects of group on the AUC of plasma glucose concentration, and glucose concentrations at the fasting state and all the sampling time points after glucose load were small and not statistically significant (*F* < 2.47, *p* > .09, *ƒ*^2^ = 0.01–0.06; Supplementary Table S1). The statistically significant main effects of day on glucose AUC and plasma glucose concentration at 120 min post-load (*F* = 3.36, *p* = .04, *ƒ*^2^ = 0.08; *F* = 9.07, *p* < .001, *ƒ*^2^ = 0.21; Figure 4A and B) pointed to a small to moderate increase in these values across study days, while the main effect of day on glucose concentration at 0 min indicated that glucose concentration at a fasting state decreased slightly during the study (*F* = 7.51, *p* = .001, *ƒ*^2^ = 0.17).

Although all the group × day interactions were small and not statistically significant for any glucose outcomes (*F* < 1.69, *p* > .16, *ƒ*^2^ = 0.01–0.08), glucose responses appeared to differ across groups. Specifically, only the variable short sleep group showed a significant and moderate increase in glucose AUC from B_2_ to M_1_5 (mean = 71.02 mmol/L/min, 95% CI = 6.54 to 135.49, *d_z_* = 0.70; Figure 4A; Supplementary Table S2), and this was primarily driven by moderate to large increases in plasma glucose concentrations at 60 min (mean = 1.00 mmol/L, 95% CI = 0.09 to 1.91, *d_z_* = 0.67) and 120 min (mean = 1.10 mmol/L, 95% CI = 0.25 to 1.91, *d_z_* = 0.99) from B_2_ (Figure 4B). On M_2_5, plasma glucose concentration at 120 min showed a small though non-significant increase from M_1_5 (mean = 0.29 mmol/L, 95% CI = −0.54 to 1.12, *d_z_* = 0.20), but importantly, remained prominently elevated from baseline (mean = 1.37 mmol/L, 95% CI = 0.61 to 2.12, *d_z_* = 1.25). The stable short sleep group showed a more modest but statistically significant increase in plasma glucose concentration at 120 min from B_2_ to M_1_5 (mean = 0.91 mmol/L, 95% CI = 0.15 to 1.66, *d_z_* = 0.39), despite no change in glucose AUC from baseline (mean = 31.90 mmol/L/min, 95% CI = −26.58 to 90.37, *d_z_* = 0.18). For this group, on M_2_5, the plasma glucose concentration at 2 h post-load did not increase further from M_1_5 (mean = −0.47 mmol/L, 95% CI = −1.19 to 0.26, *d_z_* = −0.22), and was in fact, slightly, but not significantly, elevated from the baseline value (mean = 0.44 mmol/L, 95% CI = −0.28% to 1.15, *d_z_* = 0.38). Interestingly, a moderate decrease in fasting plasma glucose concentration from B_2_ was found after sleep restriction in both the stable (mean = −0.16 mmol/L, 95% CI = −0.29 to −0.03, *d_z_* = −0.73) and the variable (mean = −0.22 mmol/L, 95% CI = −0.36 to −0.09, *d_z_* = −0.80) short sleep groups on M_1_5, and in the stable short sleep group on M_2_5 (mean = −0.16 mmol/L, 95% CI = −0.30 to −0.02, *d_z_* = −0.55). In contrast to the significant changes in the glucose concentrations of the two short sleep groups, the control group’s glucose AUC (mean = 25.75 mmol/L/min, 95% CI = −35.80 to 87.28, *d_z_* = 0.22; mean = 30.25 mmol/L/min, 95% CI = −34.28 to 94.78, *d_z_* = 0.20) and plasma glucose concentration at all sampling time points (*p* > .06, *d_z_* = −0.24 to 0.36) did not show any significant change from B_2_ to M_1_5 and M_2_5 (Figure 4A and C; Supplementary Table S2).

### Plasma insulin profile

For the AUC of plasma insulin concentration, and insulin concentrations at the fasting state and all of the post-glucose load time points, the main effects of group were not statistically significant, and the effect sizes were negligible or small (*F* < 1.38, *p* > .26, *ƒ*^2^ = 0.004–0.03; Supplementary Table S1). Two statistically significant main effects of day were found, pointing to small increases in insulin AUC and insulin concentrations at 120 min across study days (*F* = 3.65, *p* = .03, *ƒ*^2^ = 0.08; *F* = 6.01, *p* = .004, *ƒ*^2^ = 0.14; Figure 4C and D).

The small group × day interactions for all the insulin outcomes were not statistically significant (*F* < 1.76, *p* > .15, *ƒ*^2^ = 0.02–0.08; Supplementary Table S3); however, the three groups appeared to differ in their insulin responses. Specifically, only the control group showed a moderate and statistically significant elevation in insulin AUC from B_2_ to M_2_5 (mean = 2620.30 mIU/L/min, 95% CI = 654.33 to 4586.27, *d_z_* = 0.56; Figure 4C), which was mainly due to the moderate increase in plasma insulin concentration at 120 min (mean = 45.52 mIU/L, 95% CI = 16.36 to 74.68, *d_z_* = 0.62; Figure 4D). Notably, this group’s plasma insulin concentration at 120 min also increased from B_2_ to M_1_5 (mean = 32.79 mIU/L, 95% CI = 4.47 to 61.10, *d_z_* = 0.58). Insulin concentrations of the stable short sleep group also showed moderate and statistically significant increases at 120 min from B_2_ to M_1_5 (mean = 33.01 mIU/L, 95% CI = 6.10 to 59.93, *d_z_* = 0.50), and at 30 min from M_1_5 to M_2_5 (mean = 19.69 mIU/L, 95% CI = 0.94 to 38.44, *d_z_* = 0.43). The variable short sleep group did not show any statistically significant change in plasma insulin concentrations from baseline (AUC: *p* > .44, *d_z_* = 0.005–0.36; post-glucose load: *p* > .21, *d_z_* = −0.30 to 0.35). As for fasting insulin concentration, the control group showed a small increase from B_2_ to M_2_5 (mean = 1.47 mIU/L, 95% CI = 0.05 to 2.90, *d_z_* = 0.47), while a modest and statistically significant reduction from B_2_ to M_1_5 was found in the variable short sleep group (mean = −1.46 mIU/L, 95% CI = −2.91 to −0.01, *d_z_* = −0.48).

**Figure 5:**
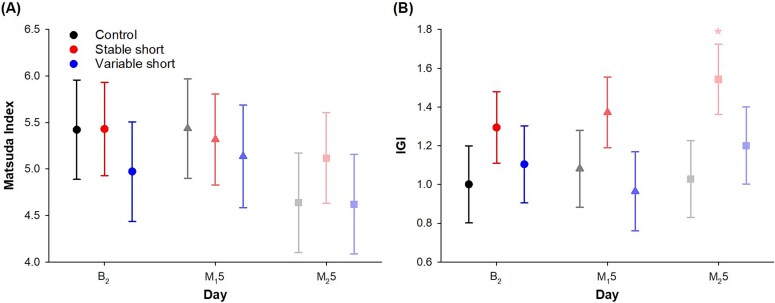
Effects of different sleep schedules on the Matsuda index and the IGI during OGTT. Least square means and standard errors of (A) Matsuda index and (B) IGI are plotted for the control group (black), the stable short sleep group (red), and the variable short sleep group (blue) on the second baseline day (B_2_ [circle]), and the day after the last sleep opportunity manipulation night in the first and second cycle (M_1_5 [triangle] and M_2_5 [square]). Asterisks indicate a significant difference from day B_2_ for each group (*p < .05).

All the main and interaction effects for the Matsuda index were not statistically significant and of negligible to small magnitude (*F* < 2.79, *p* > .07, *ƒ*^2^ = 0.004–0.06; Supplementary Table S1). All three groups did not experience any significant change in the Matsuda index during the study (*p* > .06, *d_z_* = −0.66 to 0.07; Figure 5A; Supplementary Table S4), except for the control group whose Matsuda index had a small to moderate reduction from B_2_ and M_1_5 to M_2_5 that just fell short of statistical significance (*p* = .07, *d_z_* = −0.66; *p* = .06, *d_z_* = −0.44).

Despite the small and non-significant group × day interaction (*F* = 0.99, *p* = .42, *ƒ*^2^ = 0.05; Supplementary Table S1), a moderate elevation in IGI was found from B_2_ to M_2_5 in the stable short sleep group (mean = 0.23, 95% CI = 0.04 to 0.43, *d_z_* = 0.60; Figure 5B; Supplementary Table S5), while no such change from baseline was observed in the control and the variable short sleep groups (*p* > .34, *d_z_* = −0.29 to 0.19).

## Discussion

This study has revealed relatively stable plasma glucose concentrations during OGTTs among healthy young adults whose nightly TIBs were within the age-specific recommended range. Glucose tolerance was poorer among individuals during 2 weeks of sleep restriction on simulated weekdays, regardless of the extent of variability of sleep duration across nights. Therefore, short sleep duration generally increases the risk for type 2 DM. Moreover, our data suggest that short sleep schedules that differ in how TIBs are distributed across nights may affect glucose metabolism to different extents and in different physiological pathways.

### Glucose metabolism in a well-rested state and during recurrent cycles of sleep restriction and recovery

The relatively stable plasma glucose concentrations during OGTTs shown by the control group highlight the importance of sleeping the recommended duration every night in optimizing glucose metabolism. The control group showed moderate increases in AUC and 2-h post-glucose load plasma insulin concentration, which suggests compensatory insulin hypersecretion to overcome insulin resistance. Of note, from B_2_ to M_2_5, the control group had small to moderate decreases in the Matsuda Index, which just fell short of statistical significance. As participants were not allowed to perform strenuous physical activity throughout the study, we speculate that insulin resistance developed in the control group due to the sedentary lifestyle in the laboratory [50].

Between the two short sleep groups, glucose tolerance appeared more impaired in the variable than the stable short sleep group. Firstly, while the 2-h post-glucose load plasma glucose concentrations were higher on M_1_5 than baseline for both groups, on M_2_5, it remained above the baseline level for the variable short sleep group but not for the stable short sleep group. Importantly, the increases in this marker conventionally used for DM diagnosis were of large effect sizes for the variable short sleep group (*d_z_* = 0.99–1.25), but only in the small effect size range for the stable short sleep group (*d_z_* = 0.38–0.39; Supplementary Table S3).

The two short sleep groups also differed in their insulin changes as insulin responses seemed more prominent in the stable than the variable short sleep group. Firstly, the stable short sleep group demonstrated a significant and moderate increase in plasma insulin concentration at 120 min post-load on M_1_5 (*p* = .02, *d_z_* = 0.50), while such change was of a smaller magnitude and in fact, not statistically significant in the variable short sleep group (*p* = .31, *d_z_* = 0.35). Moreover, the stable short sleep group had a more prominent acute insulin response, since it showed a significant and moderate increase in IGI from baseline to M_2_5 (*p* = .02, *d_z_* = 0.60), but this change was small and non-significant for the variable short sleep group (*p* = .34, *d_z_* = 0.19). Together with the elevated plasma glucose concentrations, the stable short sleep group showed early signs of insulin resistance. In contrast, in the variable short sleep group, plasma insulin concentration was not significantly elevated from baseline during both periods of sleep restriction despite a higher plasma glucose concentration, indicating a lack of significant compensation of insulin secretion and possibly deficits in pancreatic beta cell functions.

Our findings of poorer glucose metabolism after the first five nights of TIB curtailment to 6 h (i.e., a stable short sleep schedule) were similar to those reported in some previous studies [9, 10, 13, 18, 21]. Some studies found a reduction in both glucose tolerance and insulin sensitivity by insulin-modified intravenous glucose tolerance test in healthy young adults after four to seven nights of sleep restriction to 4 to 5 h [9, 10, 21]. Another study also reported a reduction in peripheral insulin sensitivity by hyperinsulinemic euglycemic clamp in healthy adults after their sleep had been restricted to 4 h for just one night [18]. Critically, the 2-h post-load plasma glucose concentration of the stable short sleep group at the end of the second week of sleep restriction was not statistically different from that in the first week; hence, glucose tolerance did not appear to further deteriorate upon another week of restricted sleep opportunities on simulated weekdays. However, since this study only lasted for two weeks, the possibility that glucose tolerance may worsen with more weeks of recurrent sleep restriction on weekdays cannot be ruled out. Longer studies are required to provide more comprehensive insights. In addition, dietary intake was strictly controlled in this experiment. It has been shown that sleep deprivation can disrupt the balance of hormones that regulate appetite, leading to increased hunger and cravings [51, 52]. It is possible that glucose tolerance would have been worse during recurrent sleep restriction in free-living environments.

Our findings differed from a few previous studies [17, 53]. One study did not find any change in 2-h post-load plasma glucose or insulin concentrations in OGTT after 8 weeks of sleep restriction in old long-sleepers [53]. In that study, participants’ TIB was reduced by 1.5 h from their habitual sleep, resulting in a mean TIB of 7.7 h. Glucose tolerance was not impaired in this situation, which supports the U-shaped relationship between sleep duration and risk of type 2 DM, with the lowest risk observed at 7–8 h TIB [5]. Our study supports the U-shaped relationship by showing impaired glucose tolerance at 6-h TIB. Another study also did not show any change in 2-h post-load plasma glucose or insulin concentration in OGTT after three nights of sleep restriction in healthy young adults [17]. Their participants’ TIB was reduced by 1 to 3 h from their habitual sleep, and the mean TIB after intervention was 6.0 h, similar to our stable short sleep group’s TIB of 6 h during the manipulation nights. Although in that study, plasma glucose and insulin concentration did not change, an increase in insulin AUC and a reduction in insulin sensitivity by the Matsuda index was observed. This also supports that 6-h TIB increases the risk of type 2 DM [5] by inducing insulin resistance, but it may require a longer period of sleep restriction (e.g., 10 nights in our study) for noticeable effects on glucose tolerance to manifest.

### Potential mechanisms of impaired glucose tolerance by sleep restriction

Our findings suggest that short sleep patterns that differ in TIB variability across nights reduce glucose tolerance through different physiological mechanisms. Several studies provided evidence on how sleep restriction may impair glucose tolerance through development of insulin resistance in skeletal muscles and adipose tissue [9, 14, 15, 18, 22]. Two studies showed an increase in serum fasting non-esterified fatty acid (NEFA) level after two or five nights of sleep restriction to 4 h [14, 22], while another study showed an increase in serum NEFA level during hyperinsulinemic euglycemic clamp after one night of sleep restriction at 4 h [18], which are suggestive of stimulated lipolysis after sleep restriction. As insulin resistance leads to increased levels of lipolysis [54], sleep restriction may induce insulin resistance in adipose tissue. Broussard et al. provided further evidence on reduced insulin sensitivity in adipose tissue after restricted sleep to 4.5 h for four nights by performing insulin signaling assay on subcutaneous abdominal fat tissue obtained from biopsies [9]. A previous study has shown a tendency for reduced insulin signaling in skeletal muscles after two nights of sleep restriction [15], which also points toward insulin resistance. There is also evidence that pancreatic beta cell responsivity to glucose is reduced after sleep curtailment [19, 27], which may be another mechanism contributing to reduced glucose tolerance.

Also, it has been widely hypothesized that sleep restriction may elicit a physiological stress response activating the hypothalamus-pituitary-adrenal (HPA) axis wherein exogenous administration of cortisol, which is hypersecreted when the HPA axis is activated, has been shown to induce insulin resistance [55]. Although a number of studies have found that sleep restriction does not increase overall mean cortisol [13, 18], a study by Buxton et al. found the converse [27]. This could be attributed to the requirement for their participants to increase their TIB to 10 h during the 3-week pre-study period. A number of studies have found that despite the lack of increased overall mean cortisol, cortisol levels were elevated in the later part of the day instead of the usual diurnal decline which is thought to reflect an impairment of the negative feedback control of the HPA axis and contribute to age-related insulin resistance [20].

Finally, catecholamines may also have a role to play in impairing glucose tolerance following sleep restriction. Sleep restriction to 5.5 h for 14 days has been reported to consistently give rise to a 20%–25% increase in overnight catecholamine levels, and exposure to high levels of epinephrine and norepinephrine reduced insulin sensitivity and impaired glucose tolerance [13].

### Limitations and future studies

Our study had a few limitations. Firstly, this experimental study was relatively short and investigated the impact of two weeks of curtailing sleep to an average of 6 h per simulated weeknight and 8-h recovery sleep over simulated weekends. Whether more prominent and compounding impact of recurrent sleep loss on glucose metabolism can be observed over longer periods and with different combinations of TIBs on weekdays and weekends remains to be addressed.

Secondly, we did not measure the levels of several hormones, including glucagon, cortisol, growth hormone, epinephrine, and norepinephrine, which are known to regulate blood glucose levels [56]. Therefore, we could not determine if sleep restriction and variability had any impact on the secretion and action of these hormones, thereby modulating blood glucose levels, which should be addressed in future studies.

Thirdly, only OGTT was performed in our study, but the gold standard for measuring insulin sensitivity is by hyperinsulinemic-euglycemic clamp [57]. A previous study found reduced insulin sensitivity by clamp but no change in 2-h post-glucose load plasma insulin concentration after sleep restriction [14], which also points toward the limitation of measuring insulin level in OGTT.

Fourthly, our sample consisted of healthy young adults only. Future studies should examine whether similar changes in glucose metabolism can be observed in other age groups, especially among older adults who have the highest prevalence of diabetes and tend to have shorter sleep duration [58], when they undergo multiple periods of sleep restriction that differ in TIB distributions across nights. Given our healthy sample, we would like to note that our findings may not be generalizable to individuals with other underlying risk factors for T2DM because previous studies have found that the association between short sleep duration and T2DM incidence was reduced in persons with family history for T2DM [59], and those who are obese [60]. Future studies can systematically investigate the impact of different short sleep schedules on glucose and insulin responses in individuals with and without established risk factors for T2DM.

Fifthly, while the meals provided during the study were catered to participants’ individual caloric needs and were designed to ensure no weight loss or gain, we did not assess body composition or weight to verify this. Additionally, we did not collect data on the macronutrient compositions of our participants’ habitual diets and thus could not determine whether a change to their diet macronutrient composition might have contributed to our findings.

Finally, previous studies have shown that sleep curtailment can delay melatonin secretion and hence, circadian rhythm [61, 62]. Future studies should examine the effects of different short sleep patterns on melatonin secretion and circadian rhythm, and determine whether this could be a potential pathway to impact glucose regulation. Relatedly, light can have a significant impact on glucose metabolism, particularly at night [63], which might contribute to the differences between the two short sleep groups and the control group observed in our study. While our study was not designed to investigate this possibility, it is important to note that in real-world settings, sleep curtailment is often accompanied by increased duration of light exposure. Future studies that involve TIB manipulation in dimly lit and normal lighting conditions will be needed to fully understand the contribution of light exposure to impaired glucose metabolism during sleep restriction.

## Conclusion

In summary, glucose homeostasis in healthy young adults is hampered during sleep restriction on weekdays, which may increase the risk of type 2 DM. Stable and variable short sleep schedules may reduce glucose tolerance to different extents, and through insulin resistance and impeding pancreatic beta cell functions, respectively.

## Supplementary Material

Supplementary_tables_25Oct2025_zsaf339

## Data Availability

The data underlying this article will be shared on reasonable request to the corresponding author.
