# Supplementary material for: Glucose homeostasis during recurrent periods of sleep restriction and recovery in healthy young adults
Source: Sleep. 2025 Oct 30;49(4):zsaf339. doi: 10.1093/sleep/zsaf339 (PMC13089489; doi:10.1093/sleep/zsaf339)
Supplement: Supplementary_tables_25Oct2025_zsaf339 [file supplementary_tables_25oct2025_zsaf339.docx]

**Glucose homeostasis during recurrent periods of sleep restriction and recovery in healthy young adults**

Yuki Y. Y. Cheung^1a^; Torance Y. L. Tan^1a^; Tiffany B. Koa^1^; Chin Meng Khoo^2^; June C. Lo^1,2,3^

^1^ Centre for Sleep and Cognition, Yong Loo Lin School of Medicine, National University of Singapore, Singapore

^2^ Department of Medicine, Yong Loo Lin School of Medicine, National University of Singapore, Singapore

^3^ Human Potential Translational Research Programme, Yong Loo Lin School of Medicine, National University of Singapore, Singapore

^a^ These two authors contributed equally to this manuscript.

Corresponding author:

Dr. June Chi-Yan Lo

Centre for Sleep and Cognition,

Yong Loo Lin School of Medicine,

National University of Singapore,

MD1 Tahir Foundation Building, 12 Science Drive 2,

Singapore 117549

Phone: (+65) 66016146

E-mail: june.lo@nus.edu.sg

**Table S1. Main and interaction effects of group and day on glucose and insulin outcomes.**

|  | **Group** | | | **Day** | | | **Group × day interaction** | | |
| --- | --- | --- | --- | --- | --- | --- | --- | --- | --- |
|  | ***F*** | ***p*** | ***f^2^*** | ***F*** | ***p*** | ***f^2^*** | ***F*** | ***p*** | ***f^2^*** |
| **Glucose** |  |  |  |  |  |  |  |  |  |
| AUC | 0.47 | 0.63 | 0.01 | **3.36** | **0.04** | **0.08** | 0.31 | 0.87 | 0.01 |
| 0 min | 1.99 | 0.14 | 0.05 | **7.51** | **0.001** | **0.17** | 0.86 | 0.49 | 0.04 |
| 15 min | 2.47 | 0.09 | 0.06 | 0.12 | 0.88 | 0.003 | 0.78 | 0.54 | 0.04 |
| 30 min | 1.24 | 0.29 | 0.03 | 0.60 | 0.55 | 0.01 | 1.69 | 0.16 | 0.08 |
| 60 min | 1.39 | 0.25 | 0.03 | 1.55 | 0.22 | 0.04 | 0.78 | 0.54 | 0.04 |
| 120 min | 1.27 | 0.29 | 0.03 | **9.07** | **<.001** | **0.21** | 1.24 | 0.30 | 0.06 |
| **Insulin** |  |  |  |  |  |  |  |  |  |
| AUC | 0.54 | 0.58 | 0.01 | **3.65** | **0.03** | **0.08** | 0.58 | 0.68 | 0.03 |
| 0 min | 1.28 | 0.28 | 0.03 | 2.38 | 0.10 | 0.05 | 1.76 | 0.15 | 0.08 |
| 15 min | 1.26 | 0.29 | 0.03 | 0.52 | 0.60 | 0.01 | 0.67 | 0.62 | 0.03 |
| 30 min | 1.38 | 0.26 | 0.03 | 2.83 | 0.06 | 0.07 | 1.12 | 0.35 | 0.05 |
| 60 min | 0.27 | 0.76 | 0.01 | 2.13 | 0.13 | 0.05 | 0.34 | 0.85 | 0.02 |
| 120 min | 0.23 | 0.80 | 0.01 | **6.01** | **0.004** | **0.14** | 1.52 | 0.20 | 0.07 |
| **Matsuda Index** | 0.18 | 0.84 | 0.004 | 2.79 | 0.07 | 0.06 | 0.35 | 0.84 | 0.02 |
| **Insulinogenic Index** | 1.29 | 0.28 | 0.03 | 1.79 | 0.17 | 0.04 | 0.99 | 0.42 | 0.05 |

Note: Statistically significant effects (*p* < 0.05) are indicated in bold. Effect sizes are quantified with Cohen’s *f^2^*.

**Table S2. Effects of different sleep schedules on plasma glucose levels during OGTTs**

|  | **AUC** | | | **0 min** | | | **15 min** | | | **30 min** | | | **60 min** | | | **120 min** | | |
| --- | --- | --- | --- | --- | --- | --- | --- | --- | --- | --- | --- | --- | --- | --- | --- | --- | --- | --- |
|  | **Mean** | **SEM** | **95% CI** | **Mean** | **SEM** | **95% CI** | **Mean** | **SEM** | **95% CI** | **Mean** | **SEM** | **95% CI** | **Mean** | **SEM** | **95% CI** | **Mean** | **SEM** | **95% CI** |
| **Control** |  |  |  |  |  |  |  |  |  |  |  |  |  |  |  |  |  |  |
| B_2_ | 837.25 | 28.47 | 780.65,893.85 | 4.37 | 0.09 | 4.20,4.54 | 6.24 | 0.22 | 5.80,6.68 | 7.82 | 0.27 | 7.29,8.35 | 8.37 | 0.39 | 7.58,9.15 | 5.28 | 0.32 | 4.64,5.92 |
| M_1_5 | 863.00 | 28.47 | 806.40,919.60 | 4.31 | 0.09 | 4.14,4.48 | 6.39 | 0.22 | 5.94,6.83 | 8.24 | 0.27 | 7.71,8.77 | 8.41 | 0.39 | 7.62,9.19 | 5.71 | 0.32 | 5.07,6.34 |
| M_2_5 | 867.50 | 28.47 | 810.90,924.10 | 4.32 | 0.09 | 4.15,4.49 | 6.31 | 0.22 | 5.86,6.75 | 8.35 | 0.27 | 7.82,8.87 | 8.33 | 0.39 | 7.54,9.11 | 5.93 | 0.32 | 5.30,6.57 |
| *d_z_* (B_2_ vs M_1_5) | 0.22 | | | -0.24 | | | 0.17 | | | 0.33 | | | 0.02 | | | 0.29 | | |
| *d_z_* (B_2_ vs M_2_5) | 0.20 | | | -0.24 | | | 0.09 | | | 0.35 | | | -0.02 | | | 0.36 | | |
| *d_z_* (M_1_5 vs M_2_5) | 0.04 | | | 0.04 | | | -0.14 | | | 0.10 | | | -0.05 | | | 0.15 | | |
| **Stable**  **short** |  |  |  |  |  |  |  |  |  |  |  |  |  |  |  |  |  |  |
| B_2_ | 826.19 | 27.25 | 772.02,880.35 | 4.49 | 0.08 | 4.33,4.65 | 6.60 | 0.21 | 6.17,7.02 | 8.29 | 0.25 | 7.79,8.79 | 7.50 | 0.38 | 6.75,8.25 | 5.68 | 0.31 | 5.07,6.29 |
| M_1_5 | 858.08 | 25.99 | 806.42,909.75 | 4.33 | 0.08 | 4.17,4.49 | 6.41 | 0.20 | 6.00,6.81 | 8.06 | 0.24 | 7.57,8.54 | 7.79 | 0.36 | 7.08,8.51 | 6.58 | 0.29 | 6.00,7.16 |
| M_2_5 | 862.92 | 25.99 | 811.25,914.58 | 4.33 | 0.08 | 4.17,4.48 | 6.62 | 0.20 | 6.22,7.03 | 8.47 | 0.24 | 7.99,8.95 | 7.93 | 0.36 | 7.22,8.65 | 6.12 | 0.29 | 5.54,6.70 |
| *d_z_* (B_2_ vs M_1_5) | 0.18 | | | -0.73 | | | -0.15 | | | -0.21 | | | 0.12 | | | 0.39 | | |
| *d_z_* (B_2_ vs M_2_5) | 0.35 | | | -0.55 | | | -0.04 | | | 0.18 | | | 0.30 | | | 0.38 | | |
| *d_z_* (M_1_5 vs M_2_5) | 0.03 | | | -0.02 | | | 0.22 | | | 0.43 | | | 0.07 | | | -0.22 | | |
| **Variable**  **short** |  |  |  |  |  |  |  |  |  |  |  |  |  |  |  |  |  |  |
| B_2_ | 787.10 | 28.47 | 730.50,843.70 | 4.30 | 0.09 | 4.13,4.47 | 6.09 | 0.22 | 5.65,6.54 | 7.95 | 0.27 | 7.43,8.48 | 7.35 | 0.39 | 6.57,8.14 | 5.12 | 0.32 | 4.48,5.76 |
| M_1_5 | 858.12 | 30.07 | 798.34,917.90 | 4.08 | 0.09 | 3.90,4.25 | 5.98 | 0.23 | 5.51,6.44 | 7.79 | 0.28 | 7.24,8.34 | 8.35 | 0.42 | 7.52,9.18 | 6.20 | 0.34 | 5.52,6.88 |
| M_2_5 | 835.90 | 28.47 | 779.30,892.50 | 4.17 | 0.09 | 4.00,4.34 | 5.85 | 0.22 | 5.40,6.29 | 7.69 | 0.27 | 7.17,8.22 | 7.76 | 0.39 | 6.98,8.54 | 6.49 | 0.32 | 5.85,7.12 |
| *d_z_* (B_2_ vs M_1_5) | 0.70 | | | -0.80 | | | -0.25 | | | -0.18 | | | 0.67 | | | 0.99 | | |
| *d_z_* (B_2_ vs M_2_5) | 0.71 | | | -0.38 | | | -0.37 | | | -0.36 | | | 0.41 | | | 1.25 | | |
| *d_z_* (M_1_5 vs M_2_5) | -0.17 | | | 0.46 | | | -0.17 | | | -0.06 | | | -0.34 | | | 0.20 | | |

Note: Means, SEMs (standard error of the mean), and 95% confidence intervals (CI) were derived from general linear mixed models. Effect sizes are quantified with Cohen’s *d_z_*.

**Table S3. Effects of different sleep schedules on plasma insulin levels during OGTTs**

|  | **AUC** | | | **0 min** | | | **15 min** | | | **30 min** | | | **60 min** | | | **120 min** | | |
| --- | --- | --- | --- | --- | --- | --- | --- | --- | --- | --- | --- | --- | --- | --- | --- | --- | --- | --- |
|  | **Mean** | **SEM** | **95% CI** | **Mean** | **SEM** | **95% CI** | **Mean** | **SEM** | **95% CI** | **Mean** | **SEM** | **95% CI** | **Mean** | **SEM** | **95% CI** | **Mean** | **SEM** | **95% CI** |
| **Control** |  |  |  |  |  |  |  |  |  |  |  |  |  |  |  |  |  |  |
| B_2_ | 7703.30 | 1244.11 | 5230.10, 10176 | 7.48 | 0.81 | 5.88, 9.08 | 47.97 | 8.12 | 31.83, 64.11 | 66.73 | 12.97 | 40.94, 92.53 | 88.31 | 14.40 | 59.69, 116.94 | 48.40 | 13.61 | 21.35, 75.45 |
| M_1_5 | 9327.30 | 1244.11 | 6854.10, 11800 | 7.61 | 0.81 | 6.00, 9.21 | 56.57 | 8.12 | 40.43, 72.71 | 81.99 | 12.97 | 56.2, 107.79 | 92.03 | 14.40 | 63.4, 120.65 | 81.19 | 13.61 | 54.14, 108.24 |
| M_2_5 | 10324 | 1244.11 | 7850.40, 12797 | 8.95 | 0.81 | 7.35, 10.56 | 53.45 | 8.12 | 37.31, 69.59 | 84.11 | 12.97 | 58.32, 109.91 | 105.43 | 14.40 | 76.81, 134.06 | 93.92 | 13.61 | 66.87, 120.97 |
| *d_z_* (B_2_ vs M_1_5) | 0.44 | | | 0.05 | | | 0.37 | | | 0.43 | | | 0.07 | | | 0.58 | | |
| *d_z_* (B_2_ vs M_2_5) | 0.56 | | | 0.47 | | | 0.21 | | | 0.40 | | | 0.32 | | | 0.62 | | |
| *d_z_* (M_1_5 vs M_2_5) | 0.23 | | | 0.36 | | | -0.20 | | | 0.06 | | | 0.28 | | | 0.23 | | |
| **Stable**  **short** |  |  |  |  |  |  |  |  |  |  |  |  |  |  |  |  |  |  |
| B_2_ | 9727.37 | 1167.65 | 7406.15, 12049 | 7.11 | 0.76 | 5.59, 8.62 | 60.22 | 7.68 | 44.95, 75.48 | 93.39 | 12.18 | 69.18, 117.61 | 101.55 | 13.68 | 74.36, 128.74 | 69.06 | 12.98 | 43.25, 94.86 |
| M_1_5 | 9945.13 | 1135.71 | 7687.41, 12203 | 6.22 | 0.74 | 4.76, 7.68 | 63.07 | 7.41 | 48.34, 77.81 | 92.73 | 11.84 | 69.18, 116.27 | 84.53 | 13.15 | 58.4, 110.66 | 102.07 | 12.42 | 77.37, 126.76 |
| M_2_5 | 10876 | 1135.71 | 8618.12, 13134 | 6.28 | 0.74 | 4.82, 7.75 | 71.13 | 7.41 | 56.39, 85.86 | 112.42 | 11.84 | 88.87, 135.96 | 109.88 | 13.15 | 83.75, 136.01 | 76.26 | 12.42 | 51.56, 100.95 |
| *d_z_* (B_2_ vs M_1_5) | 0.06 | | | -0.39 | | | 0.11 | | | 0.03 | | | -0.21 | | | 0.50 | | |
| *d_z_* (B_2_ vs M_2_5) | 0.23 | | | -0.30 | | | 0.11 | | | 0.42 | | | 0.12 | | | 0.16 | | |
| *d_z_* (M_1_5 vs M_2_5) | 0.22 | | | 0.02 | | | 0.19 | | | 0.43 | | | 0.42 | | | -0.34 | | |
| **Variable**  **short** |  |  |  |  |  |  |  |  |  |  |  |  |  |  |  |  |  |  |
| B_2_ | 8424.00 | 1244.11 | 5950.80, 10897 | 8.13 | 0.81 | 6.52, 9.73 | 52.83 | 8.12 | 36.69, 68.97 | 80.57 | 12.97 | 54.77, 106.36 | 85.23 | 14.40 | 56.6, 113.85 | 64.09 | 13.61 | 37.04, 91.14 |
| M_1_5 | 8452.03 | 1289.96 | 5887.68, 11016 | 6.66 | 0.82 | 5.03, 8.30 | 51.76 | 8.38 | 35.11, 68.41 | 68.48 | 13.41 | 41.83, 95.13 | 82.43 | 15.12 | 52.38, 112.49 | 79.41 | 14.33 | 50.92, 107.9 |
| M_2_5 | 9187.75 | 1244.11 | 6714.55, 11661 | 7.62 | 0.81 | 6.02, 9.22 | 50.57 | 8.12 | 34.43, 66.71 | 82.37 | 12.97 | 56.58, 108.17 | 93.23 | 14.40 | 64.6, 121.85 | 77.45 | 13.61 | 50.4, 104.5 |
| *d_z_* (B_2_ vs M_1_5) | 0.005 | | | -0.48 | | | -0.08 | | | -0.30 | | | -0.10 | | | 0.35 | | |
| *d_z_* (B_2_ vs M_2_5) | 0.36 | | | -0.20 | | | -0.07 | | | 0.04 | | | 0.20 | | | 0.35 | | |
| *d_z_* (M_1_5 vs M_2_5) | 0.30 | | | 0.39 | | | -0.06 | | | 0.49 | | | 0.31 | | | -0.01 | | |

Note: Means, SEMs (standard error of the mean), and 95% confidence intervals (CI) were derived from general linear mixed models. Effect sizes are quantified with Cohen’s *d_z_*.

**Table S4. Effects of different sleep schedules on Matsuda index**

|  | **Mean** | **SEM** | **95% CI** |
| --- | --- | --- | --- |
| **Control group** |  |  |  |
| B_2_ | 5.42 | 0.53 | 4.36,6.48 |
| M_1_5 | 5.44 | 0.53 | 4.37,6.50 |
| M_2_5 | 4.64 | 0.53 | 3.58,5.70 |
| *d_z_* (B_2_ vs M_1_5) |  | 0.01 |  |
| *d_z_* (B_2_ vs M_2_5) |  | -0.66 |  |
| *d_z_* (M_1_5 vs M_2_5) |  | -0.44 |  |
| **Stable short sleep group** |  |  |  |
| B_2_ | 5.43 | 0.50 | 4.43,6.42 |
| M_1_5 | 5.32 | 0.49 | 4.35,6.29 |
| M_2_5 | 5.12 | 0.49 | 4.15,6.10 |
| *d_z_* (B_2_ vs M_1_5) |  | -0.07 |  |
| *d_z_* (B_2_ vs M_2_5) |  | -0.12 |  |
| *d_z_* (M_1_5 vs M_2_5) |  | -0.10 |  |
| **Variable short sleep group** |  |  |  |
| B_2_ | 4.97 | 0.53 | 3.91.6.04 |
| M_1_5 | 5.14 | 0.55 | 4.04,6.23 |
| M_2_5 | 4.62 | 0.53 | 3.56,5.68 |
| *d_z_* (B_2_ vs M_1_5) |  | 0.07 |  |
| *d_z_* (B_2_ vs M_2_5) |  | -0.25 |  |
| *d_z_* (M_1_5 vs M_2_5) |  | -0.38 |  |

Note: Means, SEMs (standard error of the mean), and 95% confidence intervals (CI) were derived from general linear mixed models. Effect sizes are quantified with Cohen’s *d_z_*.

**Table S5. Effects of different sleep schedules on Insulinogenic index**

|  | **Mean** | **SEM** | **95% CI** |
| --- | --- | --- | --- |
| **Control group** |  |  |  |
| B_2_ | 1.00 | 0.20 | 0.60,1.40 |
| M_1_5 | 1.08 | 0.20 | 0.69,1.48 |
| M_2_5 | 1.03 | 0.20 | 0.63,1.42 |
| *d_z_* (B_2_ vs M_1_5) |  | 0.17 |  |
| *d_z_* (B_2_ vs M_2_5) |  | 0.08 |  |
| *d_z_* (M_1_5 vs M_2_5) |  | -0.14 |  |
| **Stable short sleep group** |  |  |  |
| B_2_ | 1.29 | 0.19 | 0.93,1.66 |
| M_1_5 | 1.37 | 0.18 | 1.01,1.73 |
| M_2_5 | 1.54 | 0.18 | 1.18,1.90 |
| *d_z_* (B_2_ vs M_1_5) |  | 0.16 |  |
| *d_z_* (B_2_ vs M_2_5) |  | 0.60 |  |
| *d_z_* (M_1_5 vs M_2_5) |  | 0.22 |  |
| **Variable short sleep group** |  |  |  |
| B_2_ | 1.10 | 0.20 | 0.71,1.50 |
| M_1_5 | 0.96 | 0.20 | 0.56,1.37 |
| M_2_5 | 1.20 | 0.20 | 0.81,1.60 |
| *d_z_* (B_2_ vs M_1_5) |  | -0.29 |  |
| *d_z_* (B_2_ vs M_2_5) |  | 0.19 |  |
| *d_z_* (M_1_5 vs M_2_5) |  | 0.63 |  |

Note: Means, SEMs (standard error of the mean), and 95% confidence intervals (CI) were derived from general linear mixed models. Effect sizes are quantified with Cohen’s *d_z_*.
